# In Vitro Evaluation of Antimicrobial Effects of Endodontic Irrigants Containing Disodium Edetate and Chlorhexidine Gluconate, Octenidine Dihydrochloride, and Benzalkonium Bromide Against Intracanal *Enterococcus faecalis*

**DOI:** 10.3390/jcm14197100

**Published:** 2025-10-08

**Authors:** Anna Siemińska, Katarzyna Kot, Ewa Marek, Agnieszka Chamarczuk, Magdalena Kaczała, Joanna Rasławska-Socha, Laurentia Schuster, Till Dammaschke, Liliana Szyszka-Sommerfeld, Mariusz Lipski

**Affiliations:** 1 Private Dental Practice, Klemensa Janickiego 16A, 71-270 Szczecin, Poland; ania@gmerek.eu; 2 Department of Preclinical Conservative Dentistry and Preclinical Endodontics, Pomeranian Medical University in Szczecin, Al. Powst. Wlkp. 72, 70-111 Szczecin, Poland; fantom@pum.edu.pl (K.K.); ewa.marek@pum.edu.pl (E.M.); agnieszka.chamarczuk@pum.edu.pl (A.C.); joanna.raslawska.socha@pum.edu.pl (J.R.-S.); mariusz.lipski@pum.edu.pl (M.L.); 3 Private Dental Practice, Energetyków 14C, 74-100 Gryfino, Poland; ipdmkaczala@gmail.com; 4 Department of Periodontology and Operative Dentistry, University of Münster, Waldeyerstraße 30, 48149 Münster, Germany; laurentia.schuster@ukmuenster.de (L.S.); tillda@uni-muenster.de (T.D.); 5 Laboratory for Propaedeutics of Orthodontics and Facial Congenital Defects, Chair of Maxillofacial Orthopaedics and Orthodontics, Pomeranian Medical University in Szczecin, Al. Powst. Wlkp. 72, 70-111 Szczecin, Poland

**Keywords:** anti-infective agents: *Enterococcus faecalis*, chlorhexidine, benzalkonium bromide, edetic acid, sodium hypochlorite, root canal irrigants

## Abstract

**Background/Objectives**: The objective of this in vitro study was to compare and evaluate the in vitro antimicrobial effectiveness of Endosal, Octenisolv, and Endoxal against intracanal *Enterococcus faecalis*. **Methods**: The study sample consisted of 84 extracted single-rooted human teeth, which were divided into seven groups (12 roots in each group): Group 1—Endoxal, Group 2—Octenisolv, Group 3—Endosal, Group 4—15% ethylenediaminetetraacetic acid (EDTA), Group 5—2% sodium hypochlorite (NaOCl), Group 6—0.9% sterile saline solution (NaCl), and one positive control group where no irrigant was used. The roots were sterilized within an autoclave for 30 min at 121 °C and then contaminated with *E. faecalis* bacteria, after instrumentation and removing the smear layer from canals. The root canals were irrigated using a side-vented needle, and then ISO size 40 H-file was used to obtain fine dentine chips. Aliquots taken from the canals were plated on blood agar broth and the plates were incubated for 36 h. **Results**: In this study, significant differences were observed between the antimicrobial activity of Endoxal, Octenisolv, Endosal, 2% NaOCl, and sterile saline solution. **Conclusions**: The compound irrigants Endosal, Endoxal, and a novel irrigant containing disodium edetate and octenidine, which were evaluated in this study, exhibited relatively good antimicrobial properties against *Enterococcus faecalis*. The use of Endosal, Octenisolv or Endoxal appears promising, yet their clinical efficacy remains to be confirmed through further studies.

## 1. Introduction

Successful endodontic treatment is primarily reliant on the effective elimination of microbial pathogens from the intricate and often inaccessible areas of the root canal system [1]. Intracanal bacteria are usually detected as sessile multispecies communities (biofilms) colonizing the dentinal root canal walls [2]. Although more than 500 bacterial species have been identified in endodontic diseases, only 20–30 are commonly found [2]. Most anaerobic bacteria are readily eliminated during root canal therapy; however, facultative bacteria often persist despite disinfection efforts [3]. Among these, *Enterococcus faecalis* is the most commonly isolated or detected species from oral infections, including marginal periodontitis [4] and infected root canals, especially with chronic apical periodontitis [5]. *E. faecalis* survives in a higher prevalence than other endodontic taxa [6]. It has been suggested that *E. faecalis* virulence may be related to its resistance to intracanal medicaments; its ability to penetrate dentinal tubules, sometimes to a deep extent; its adaptation to varying environmental conditions by regulation of gene expression; and an ability to survive in the root canal as a single organism without the support of other bacteria [7,8,9]. Mechanical instrumentation of the root canal removes the majority of microorganisms; however, it is not sufficient, because of the complex and unpredictable anatomy of the root canal system. Peters et al. [10] showed that mechanical preparation can leave up to 40–50% surface of the canal walls uninstrumented. The effectiveness of mechanical instrumentation of the root canal can be improved by using suitable irrigants. Chemical preparation by using irrigation solutions helps to clean those areas of the root canal that could be inaccessible for endodontic instruments [11]. Irrigants provide lubrication, debridement, an antimicrobial effect, and the dissolution of organic and inorganic material [12]. Considering these limitations, and to simplify irrigation protocol, new combinational products have been introduced as the final irrigants, after sodium hypochlorite (NaOCl). BioPure MTAD (Dentsply, Tulsa Dental, Tulsa, OK, USA) has recently been developed for smear layer removal and canal disinfection. BioPure MTAD is a mixture of a tetracycline (doxycycline), an acid (citric acid), and a detergent (Tween 80) [13,14,15]. Tetraclean (Ogna Laboratori Farmaceutici, Muggio, Italy), like MTAD, is a mixture of an antibiotic, an acid, and a detergent. However, the concentration of the antibiotic, doxycycline (lower concentration), and the type of detergent (polypropylene glycol) differ from those of MTAD [16,17,18]. Doxycycline, the main component of MTAD, absorbs strongly to tooth surfaces, providing prolonged antibacterial activity. However, one study reported discolouration of root dentin when 1.3% NaOCl was used prior to MTAD irrigation [19]. Another study demonstrated that while MTAD is less toxic than 5.25% NaOCl, it exhibits greater toxicity when compared to NaOCl at reduced concentrations of 2.63%, 1.33%, and 0.66% [20]. Due to concerns over the use of antibiotic-based root canal irrigants, such as the risks of bacterial resistance, allergic reactions, inhibition of angiogenesis, and tooth discoloration, there is growing interest in antibiotic-free alternatives [21,22]. Other compound irrigant solutions commonly used in endodontics but containing ethylenediaminetetraacetic acid (EDTA), and chlorhexidine (CHX), are QMix 2in1 (Dentsply Tulsa Dental, Tulsa, OK, USA), SmearOFF (Vista Dental Produkts, Racine, WI, USA) and Endoxal (0.2% chlorhexidine gluconate, Chema-Elektromet, Rzeszów, Poland) [23,24,25]. A well-known disinfectant in medical facilities, which also demonstrated promising results in eradicating root canal infections, is octenidine (OCT) [26,27]. Octenisolv (Chema-Elektromet, Rzeszów, Poland) is an experimental solution that has been created for the needs of the current study. It consists of 15% EDTA, 0.1% octenidine dihydrochloride, sodium hydroxide, and purified water. A different irrigant solution with similar composition to Octenisolv is Endosal (Chema-Elektromet, Rzeszów, Poland). Endosal contains a disinfecting agent in the form of benzalkonium bromide (0.76%), which also acts as a detergent.

The aim of this in vitro study was to evaluate the antimicrobial effects of final irrigation with endodontic irrigants containing disodium edetate and chlorhexidine gluconate (Endoxal), octenidine dihydrochloride (Octenisolv), and benzalkonium bromide (Endosal) against intracanal *Enterococcus faecalis* (absolute CFU reduction vs. log-reduction). This study had three null hypotheses. The first hypothesis was that there were no differences in the disinfection efficacy of irrigants containing EDTA and chlorhexidine gluconate (Endoxal), octenidine dihydrochloride (Octenisolv), and benzalkonium bromide (Endosal) in root canals infected with Enterococcus faecalis. The second hypothesis was that an irrigant containing only EDTA was less effective for eradicating *E. faecalis* from extracted human teeth than irrigants containing EDTA and chlorhexidine gluconate, octenidine dihydrochloride, and benzalkonium bromide. The third hypothesis was that sodium hypochlorite eliminated *E. faecalis* more effectively than an irrigant containing only EDTA and irrigants containing EDTA and chlorhexidine gluconate, octenidine dihydrochloride, or benzalkonium bromide.

## 2. Materials and Methods

### 2.1. Sample Size

To detect true differences between 8 groups of teeth with effect size corresponding to RMSSE (Root Mean Square Standardized Effect) 0.5, alpha level 0.05 and statistical power 90%, the number of teeth needed in each group is 12.

### 2.2. Specimen Preparations

Eighty-four single-rooted extracted human teeth without caries, with completely developed roots, were used in the present study. The study protocol was approved by the Ethics Committee of Pomeranian Medical University of Szczecin (KB-0012/10/19). All teeth were extracted because of periodontal or orthodontic reasons. After cleaning the root surface with periodontal curettes, all teeth were immersed in 2% sodium hypochlorite (NaOCl) for 2 h to remove organic material from the root surface. The teeth were decoronated at the cemento-enamel junction, using a high-speed diamond bur with copious water cooling to standardize the root length at 12 mm. A size 10 K-file was inserted into each canal until its tip was visible at the apical foramen, and the length of the canal was measured. The working length was established by subtracting 1 mm from the measured length. Root canal instrumentation was performed by using Gates Glidden burs to ISO size 50 (VDW; Munich, Germany). Subsequently, apical preparation was checked with hand K-file size 50. The canals were irrigated with 2 mL of 2% sodium hypochlorite (Chloraxid 2%; Cerkamed, Stalowa Wola, Poland) after each instrument, using a 30 G side-vented needle (Endo-Top; Cerkamed, Stalowa Wola, Poland) and a syringe. The needle tip was positioned approximately 1.5 mm shorter than working length. To enhance irrigant activation, a gentle up-and-down motion was performed during the irrigation. After chemo-mechanical preparation, the smear layer was removed with 1 mL of 17% EDTA (Cerkamed; Stalowa Wola, Poland), followed by 5 mL of sterile saline solution. The canals were dried with sterile absorbent paper points (Sure-Endo, Sangdewon-dong, Seongnam-si, Republic of Korea), the roots were coated with 2 coats of nail varnish, and apical foramens were sealed with light-cured composite resin (Boston, Arkona, Lublin, Poland). To eliminate the variability, the preparation of the samples was performed by the same operator. The specimens were sterilized by autoclave for 30 min at 121 °C to kill microorganisms inside the roots.

After sterilization, the teeth were submitted to the sterilization control. A sterile paper point was placed in contact with the root canal walls for 15 s, individually transported to a plastic microtube containing 1 mL of BHI broth, and vortexed for 1 min. Next, 0.1 mL of broth from each tube was plated onto separate BHI + blood agar plates and incubated at 37 °C for 48 h. Since all samples showed no bacterial growth, all teeth qualified for the study.

### 2.3. Bacterial Strains and Growth Conditions

The suspension culture of *E. faecalis* (ATCC 29212) was prepared in tryptic soy broth (TSB) (Difico; Detroit, MI, USA). TSB was incubated for 24 h to obtain the density of microorganisms equal to 0.5 McFarland constant, which is equivalent to 1.5 × 10^8^ colony forming units—CFU/mL. The bacterial suspension was introduced into root canals to obtain biofilm formation. Then, samples were incubated at 37 °C for 7 days. Freshly made suspension was injected into root canals after 24, 94, and 144 h. After incubation, roots were randomly divided into 6 experimental groups (*n* = 12 roots on each), according to the types of irrigant used as the final rinse and positive control groups (*n* = 12 roots). For randomization, a general dentist not participating in the study chose one tooth from the database of prepared samples and assigned it to Group 1. The next selected tooth from the sample database was assigned to Group 2, and the next teeth were assigned to subsequent groups. After assigning one tooth to each of the 7 groups (6 experimental and positive control), the procedure was repeated, starting from Group 1 and ending with Group 7. The randomization was completed when 12 teeth were assigned to each group and no teeth remained in the database. Depending on the group, the teeth were assigned a sequential number from 1 to 12 (e.g., 1.1 to 1.12 for samples from Group 1; 2.1 to 2.12 for Group 2, etc.).

The irrigation protocol, rising for 1.5 min was as follows:

- Group 1 (*n* = 12): 2 mL of Endoxal (Chema-Elektromet, Rzeszów, Poland);
- Group 2 (*n* = 12): 2 mL of Octenisolv (Chema-Elektromet, Rzeszów, Poland);
- Group 3 (*n* = 12): 2 mL of Endosal (Chema-Elektromet, Rzeszów, Poland);
- Group 4 (*n* = 12): 2 mL of a 15% EDTA (Chema-Elektromet, Rzeszów, Poland);
- Group 5 (*n* = 12): 2 mL of 2% NaOCl (Chloraxid 2.0%; Cerkamed, Stalowa Wola, Poland);
- Group 6 (*n* = 12): 2 mL of sterile saline solution (0.9% NaCl);
- Group 7 (*n* = 12): no decontamination procedure was performed (positive control group).

For every irrigation, a syringe and 30 G side-vented needle were used. During irrigation, the needle was inserted 1.5 mm shorter than working length and moved gently with an up-and-down motion. To prevent prolonged action of irrigating solutions, 1 mL of a neutralizing substance was applied in each root canal. Irrigant agents and neutralizing substances are presented in Table 1. The solutions containing neutralizing substances were slowly applied to the canals, ensuring contact with the wall for at least two minutes. Next, the canals were then dried with sterile paper points and irrigated with 30 mL sterile saline solution to prevent potential carry-over of the irrigants. Canals were dried again with sterile absorbent paper points. A total of 8 μL of sterile saline solution was introduced into the canal and ISO size 40 H-file was used to obtain fine dentine chips.

A 1 μL inoculation loop was used to remove aliquots from the fluid in the root canal system. The aliquots were plated on blood agar broth (BioMerieux, Craponne, France) and the plates were incubated for 36 h. The number of colony forming units (CFU) of *E. faecalis* served as a measure of antimicrobial activity. In the case of 15% EDTA and sterile saline solution, additional dilution was used (1:10).

The numbers of CFUs were determined traditionally. The counting was performed 3 times in each plate by an observer, which was previously calibrated. A second observer performed the same procedure in order to confirm the counting of the number of CFUs. If individual colonies could not be discriminated on a plate, the plate with the higher dilution was considered for evaluation. The observers had no information regarding the irrigant and dilution used.

### 2.4. Statistical Analysis

Descriptive statistics, including mean value, standard deviation, median, interquartile range (IQR), and minimum and maximum values, were calculated as CFU for antibacterial effects of all evaluated solutions. Log-transformed CFU value was calculated as Log_10_(CFU + 1), yielding zero value for CFU = 0. The normality of the data was calculated using the Shapiro–Wilk test. Since the CFU data were not normally distributed, the Kruskal–Wallis test with Siegel–Castellan post hoc procedure was used. The significance level was set at *p* < 0.05. Statistical calculations were performed with Statistica 13 software.

## 3. Results

**Figure 1:**
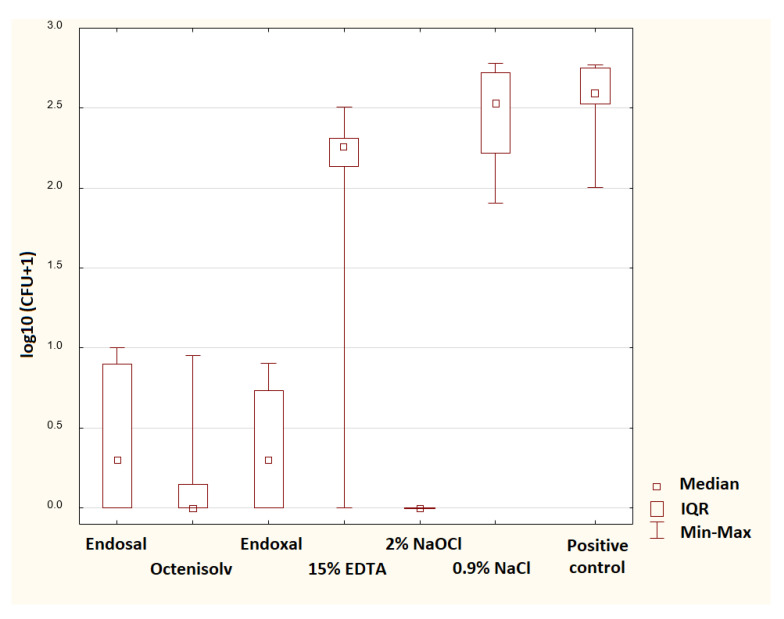
Log-transformed CFU values of *E. faecalis*.

The CFU of *E. faecalis* after rinsing with the irrigant solutions are presented in Table 2 and Table 3. The most effective irrigant against *E. faecalis* was 2% sodium hypochlorite. No bacterial growth was observed in all samples irrigated with NaOCl. Relatively good antimicrobial properties were also observed for Endoxal, Octenisolv and Endosal. No statistically significant difference was detected when comparing these combined preparations with 2% NaOCl (Table 4). The 15% EDTA showed a worse ability to eliminate the *E. faecalis* compared to sodium hypochlorite and the irrigant containing disodium edetate and octenidine, and sterile saline solution was the least effective solution (statistically significant difference compared to all solutions evaluated except EDTA, Table 4). The log-transformed CFU values of *E. faecalis* are visualized in Figure 1.

**Figure 2:**
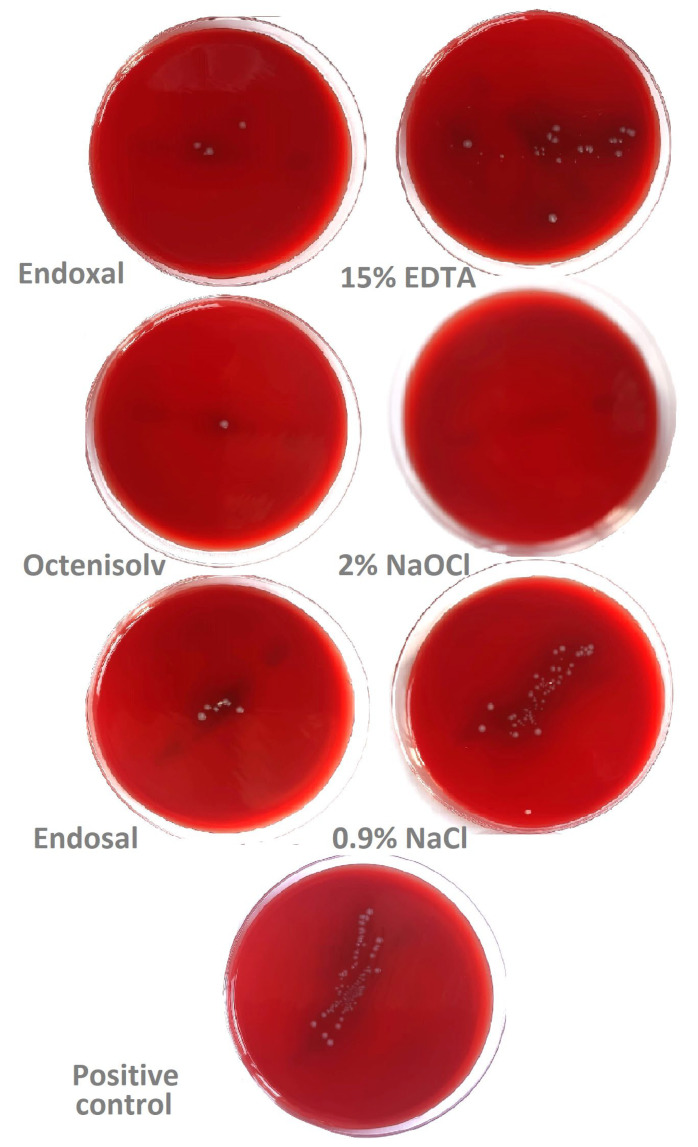
Colony growth of *Enterococcus faecalis* on blood agar broth plates after 36 h incubation at 37 °C. In the case of 15% EDTA, 0.9% NaCl and positive control, a dilution of 1:10 was used.

Figure 2 presents the colony growth of *Enterococcus faecalis* on blood agar broth plates after 36 h incubation at 37 °C.

## 4. Discussion

This study had three null hypotheses. The first hypothesis was that there were no differences in the disinfection efficacy of irrigants containing EDTA and chlorhexidine gluconate (Endoxal), octenidine dihydrochloride (Octenisolv), and benzalkonium bromide (Endosal) in root canals infected with *Enterococcus faecalis*. No statistically significant difference was detected when comparing Endoxal, Octenisolv, and Endosal. Therefore, the first research hypothesis was accepted.

The second hypothesis was that an irrigant containing only EDTA was less effective for eradicating *E. faecalis* from extracted human teeth compared to irrigants containing EDTA and chlorhexidine gluconate, octenidine dihydrochloride, and benzalkonium bromide. Statistical analysis showed a significant difference only for Octenisolv and 15% EDTA; for the other two complex formulations, no significant differences were found compared to 15% EDTA. Therefore, the second research hypothesis was partially accepted.

The third hypothesis was that sodium hypochlorite eliminated E. faecalis more effectively than an irrigant containing only EDTA and the irrigants containing EDTA and chlorhexidine gluconate, octenidine dihydrochloride, or benzalkonium bromide. This hypothesis was also partially accepted. E. faecalis was eliminated significantly more effectively with 2% NaOCl than with 15% EDTA. However, no statistically significant differences were found when sodium hypochlorite was compared to Endoxal, Octenisolv, or Endosal.

The aim of endodontic treatment is the elimination of microorganisms from the root space and prevention of reinfection. Bacteria and their by-products play a central role in the pathogenesis of pulpal necrosis and apical lesions. While necrotic root canals are generally infected by a diverse range of microorganisms—including both Gram-positive and Gram-negative bacteria in nearly equal proportions—and are predominantly anaerobic, the microbial profile in retreatment cases tends to be less complex. These infections are often monomicrobial, primarily involving Gram-positive bacteria, with facultative and obligate anaerobes occurring in similar amounts. Among these, *Enterococcus faecalis* is frequently identified as a major pathogen in teeth where root canal therapy has failed [28]. It is one of the most resistant pathogens in the root canal space due to its unique survival strategies, like the following abilities: colonizing the dentine tubules, changing the host defenses, producing lytic enzymes, and forming solid biofilm [29,30]. Thus, this research—along with numerous similar studies—has focused on the eradication of *E. faecalis* [31,32,33]. Furthermore, the pathogenic role of the other resistant microorganism should not be ignored [29].

The emergence of resistance to commonly used medications has prompted manufacturers to formulate alternative root canal irrigants. Antimicrobial effectiveness is surely the most important property required for an irrigant solution to be used during root canal treatment.

Sodium hypochlorite (NaOCl) in concentrations from 0.5% to 8.25% is the most studied root canal irrigant in endodontics. It has a broad spectrum of antimicrobial activity and dissolves the necrotic pulp tissue and organic debris [27,34,35]. Torabinejad et al. compared the bactericidal effect of 5.25% NaOCl, 17% EDTA and BioPure MTAD on *E. faecalis* [36]. In this study, the zones of inhibition and minimum inhibitory concentrations for these solutions were measured. The results showed that BioPure MTAD was as effective as 5.25% NaOCl, and significantly more effective than EDTA. However, an alternative study using an *Enterococcus faecalis* biofilm model demonstrated that BioPure MTAD was much less effective than NaOCl [18,21]. Among the tested irrigants, only 5.25% NaOCl was able to eliminate the biofilm within 5 min. In contrast, Tetraclean achieved a similar effect after 60 min, while BioPure MTAD failed to achieve complete biofilm removal at any of the evaluated time points [18]. Considering the other adverse effects associated with the use of antibiotic-containing irrigants—such as the development of bacterial resistance—the superiority of antibiotic-free solutions should be recognized [21,22]. These findings are in agreement with another study that has demonstrated the antibacterial effectiveness of NaOCl [37]. A dilution of NaOCl to 0.000625% concentration was able to eradicate biofilms of *E. faecalis* after 1 min of exposure, whereas the CHX solution was determined to be less effective, requiring at least 5 min of contact time in concentrations of 2%. At any dilution or time tested, 17% EDTA and 25% citric acid and 5% phosphoric acid solutions were not effective against *E. faecalis* [37]. Nascimento et al. [31] evaluated the antimicrobial activity of 2.5% NaOCl and 2% CHX, 0.2% cetrimide CTR, QMiX and 0.85% NaCl and 2,5% NaOCl associated with 0.2% cetrimide (CTR), and 2% CHX associated with 0.2% CTR against biofilm and planktonic *E. faecalis*. All irrigants have effectively eliminated the planktonic cells after 1 and 3 min of direct contact. Only the NaOCl solutions, alone and associated with CTR, were able to eliminate microbial biofilm. CHX showed similar antimicrobial potential to CHX + CTR, and QMix after 1 min of direct contact, and was similar to NaOCl alone and NaOCl + CTR after 3 min of direct contact. After 3 min of contact, QMix showed lower activity against *E. faecalis* biofilm than NaOCl, NaOCl + CTR, CHX, and CHX + CTR [29]. The results of our study confirmed the effectiveness of 2% NaOCl against *E. faecalis*, similarly to the findings reported by Nascimento et al. [31].

Octenidine hydrochloride (OCT) belongs to the bipyridines and has been suggested as an alternative endodontic irrigant, based on its antimicrobial effects and lower cytotoxicity [32,33]. Octenidine shows properties of positively charged chemical compounds; exhibits broad spectrum antimicrobial effects on Gram-positive and Gram-negative bacteria, fungi and several viruses; and has a specific ability to adhere to and form complexes with chemical components of cells [38,39]. OCT (0.10%) showed results that were equally as good as 2% CHX against *C. albicans*, but its bactericidal activity against *E. faecalis* was lower than 5.25% NaOCl [40]. Chum et al. [41] showed that 0.1% OCT has a comparable antimicrobial effect with 2% CHX and 3% NaOCl against *S. epidermidis*. Bukhary et al. [42] evaluated the antibacterial effectiveness of Octenisept, 1% alexidine (ALX), and 2% chlorhexidine against *E. faecalis* biofilm by using confocal laser scanning microscopy. The results showed that OCT killed the majority of *E. faecalis* in biofilm. The confocal laser scanning microscopic images of samples treated with 5.25% NaOCl showed almost complete biofilm removal from the dentin surface. OCT was more effective than CHX and ALX. In the present study, the new endodontic irrigant containing disodium edetate and octenidine showed good antimicrobial activity against *E. faecalis* after 1.5 min of irrigation, though this was statistically insignificant when compared to 2% NaOCl.

Endoxal is a premixed dual-action irrigating solution used as a final rinse after NaOCl in endodontic therapy. It is a mixture of a chlorhexidine gluconate and EDTA. Endoxal has a similar composition to the well-studied QMix [43,44,45]. Stojicič et al. [46] showed that QMix was superior to 1% NaOCl, 2% CHX and MTAD by killing *E. faecalis* and plaque bacteria in planktonic and biofilm culture. Wang et al. [47] concluded that QMix and 6% NaOCl had stronger antibacterial effects against young and old *E. faecalis* biofilms in dentin than 2% NaOCl and 2% CHX. The results of our study demonstrate that Endoxal irrigant, similar in composition to QMix, has a good effect against *E. faecalis*.

Our study also evaluated the antimicrobial effect of 15% EDTA and found it to have very limited antibacterial properties against *E. faecalis*, which is consistent with previous studies [34,48]. On the other hand, according to the studies evaluating the antifungal effect, the EDTA solution has some antifungal properties, which is due to the fact that this chelator removes calcium ions from the cell walls, causing collapses in the cell wall [49].

The sequence used in this study (chemo-mechanical preparation with NaOCl and EDTA, sterilization, contamination, final rinse testing) creates a dentine condition that may not mimic clinical biofilm ecology during actual treatment (smear layer status, prior chemical exposure, tubule content). This may impact the obtained results, and requires the creation of a model that better reflects the clinical situation.

Currently, sodium hypochlorite is the best irrigant for root canals; besides antibiofilm potency, it has ability to dissolve organic tissues [43,50,51,52,53,54]. However, it has high toxicity, can cause allergic reactions, is ineffective at removing the smear layer, and has an unpleasant taste and odor [55,56,57,58]. Endoxal, Endosal, and Octenisolv demonstrated relatively good antibacterial effect against *E. faecalis* in this study, although they did not completely eliminate this microorganism from the canal as NaOCl did. Further investigations are required to determine the antibacterial potential of these alternative irrigants in a multi-species biofilm model.

## 5. Conclusions

The compound irrigants Endoxal, Endosal, and especially a novel irrigant containing disodium edetate and octenidine, which were evaluated in this study in comparison to NaOCl and EDTA, appear to be a relatively effective final rinse solution for the eradication of *E. faecalis* from extracted human teeth. Further studies are needed to determine the effect of these findings in clinical settings.

## Figures and Tables

**Table 1:** Irrigating solutions and their neutralizing substances.

Irrigating Solution	Neutralizing Substance
Endoxal	3% detergent Tween 80 + 0.3% lecithin solution
Octenisolv	3% detergent Tween 80 + 0.3% lecithin solution + 0.1% cysteine solution
Endosal	3% detergent Tween 80 + 0.3% lecithin solution
15% EDTA	3% detergent Tween 80 + 0.3% lecithin solution
2% NaOCl	5% sodium thiosulfate solution

EDTA—ethylenediaminetetraacetic acid; NaOCl—sodium hypochlorite.

**Table 2:** The mean growth of CFU of *E. faecalis*.

Group	*n*	Mean	Median	Min	Max	25Q	75Q	IQR	SD	Normality Tests	
										W	*p*
1 (Endoxal)	12	2.25	1	0	7	0	4.5	4.5	2.63	0.821164	0.016472
2 (Octenisolv)	12	1.33	0	0	8	8	0.5	0.5	2.90	0.520059	2.49 × 10^−5^
3 (Endosal)	12	3.42	1.5	0	9	0	7	7	3.87	0.776942	0.005184
4 (15% EDTA)	12	167.5	180	0	320	135	205	70	78.75	0.94691	0.592346
5 (2% NaOCl)	12	0	0	0	0	0	0	0	0		
6 (0.9% NaCl)	12	350.83	335	80	600	165	530	365	190	0.913577	0.237043
7 (positive control)	12	409.17	390	100	590	335	565	230	162.79	0.901352	0.165119

EDTA—ethylenediaminetetraacetic acid; NaOCl—sodium hypochlorite; 0.9% NaCl—sterile saline solution; *n*—number; CFU—colony forming units; SD—standard deviation; Min—minimum; Max—maximum; 25Q—first quartile; 75Q—third quartile; IQR—interquartile range.

**Table 3:** Log-transformed CFU values of *E. faecalis*.

Group	n	Mean	Median	Min	Max	25Q	75Q	IQR	SD	Normality Tests	
										W	*p*
1 (Endoxal)	12	0.37	0.30	0	0.90	0	0.74	0.74	0.37	0.825273	0.018422
2 (Octenisolv)	12	0.18	0	0	0.95	0	0.15	0.15	0.36	0.56	4.95 × 10^−5^
3 (Endosal)	12	0.44	0.30	0	1	0	0.90	0.90	0.47	0.736432	0.001936
4 (15% EDTA)	12	2.05	2.26	0	2.51	2.13	2.31	0.18	0.66	0.530203	2.98 × 10^−5^
5 (2% NaOCl)	12	0	0	0	0	0	0	0	0		
6 (0.9% NaCl)	12	2.47	2.53	1.91	2.78	2.22	2.73	0.51	0.30	0.886323	0.105663
7 (positive control)	12	2.57	2.59	2	2.77	2.52	2.75	0.23	0.24	0.814518	0.013767

EDTA—ethylenediaminetetraacetic acid; NaOCl—sodium hypochlorite; 0.9% NaCl—sterile saline solution; *n*—number; CFU—colony forming units; SD—standard deviation; Min—minimum; Max—maximum; 25Q—first quartile; 75Q—third quartile; IQR—interquartile range.

**Table 4:** Statistical differences between bactericidal properties of tested irrigants.

Dependent: CFU	*p*-Value for Multiple Comparisons (Two-Sided): CFU						
	1 (Endoxal)	2 (Octenisolv)	3 (Endosal)	4 (15% EDTA)	5 (2% NaOCl)	6 (0.9% NaCl)	7 (Positive Control)
1 (Endoxal)		1	1	0.35	1	0.01	0.002192
2 (Octenisolv)	1		1	0.03	1	0.0004	0.000074
3 (Endosal)	1	1		0.37	1	0.01	0.002439
4 (15% EDTA)	0.35	0.03	0.37		0.002	1	1
5 (2% NaOCl)	1	1	1	0.002		0.00001	0.000002
6 (0.9% NaCl)	0.01	0.0004	0.01	1	0.00001		1
7 (positive control)	0.002	0.0001	0.002	1	0.000002	1	

EDTA—ethylenediaminetetraacetic acid; NaOCl—sodium hypochlorite; 0.9% NaCl—sterile saline solution; CFU—colony forming units.

## Data Availability

All data generated or analyzed during this study are included in this published article. Further inquiries should be directed to the corresponding author.

## Abbreviations

The following abbreviations are used in this manuscript:

ALX	alexidine
CFU	colony forming units
CHX	bisbiguanide antimicriobial agent—chlorhexidine
CTR	cetrimide
EDTA	ethylenediaminetetraacetic acid
NaOCl	sodium hypochlorite
MTAD	mixture of a tetracycline, an acid, and a detergent
0.9% NaCl	sterile sodium solution
OCT	octenidine hydrochloride
